# Is There a Single Ideal Parameter for Halogen‐Bonding‐Based Lewis Acidity?

**DOI:** 10.1002/chem.201905273

**Published:** 2020-03-09

**Authors:** Elric Engelage, Dominik Reinhard, Stefan M. Huber

**Affiliations:** ^1^ Organische Chemie I Fakultät für Chemie und Biochemie Ruhr-Universität Bochum Universitätsstraße 150 44801 Bochum Germany

**Keywords:** bond energy, density functional calculations, halogen bonding, iodine, Lewis acids

## Abstract

Halogen‐bond donors (halogen‐based Lewis acids) have now found various applications in diverse fields of chemistry. The goal of this study was to identify a parameter obtainable from a single DFT calculation that reliably describes halogen‐bonding strength (Lewis acidity). First, several DFT methods were benchmarked against the CCSD(T) CBS binding data of complexes of 17 carbon‐based halogen‐bond donors with chloride and ammonia as representative Lewis bases, which revealed M05‐2X with a partially augmented def2‐TZVP(D) basis set as the best model chemistry. The best single parameter to predict halogen‐bonding strengths was the static σ‐hole depth, but it still provided inaccurate predictions for a series of compounds. Thus, a more reliable parameter, *Ω*_σ*_, has been developed through the linear combination of the σ‐hole depth and the σ*(C−I) energy, which was further validated against neutral, cationic, halogen‐ and nitrogen‐based halogen‐bond donors with very good performance.

## Graphical abstract

**Inspecting halogen bonding**: Is it possible to predict halogen‐bonding strength accurately by a single DFT calculation and achieve better performance than that offered by the static σ‐hole depth?

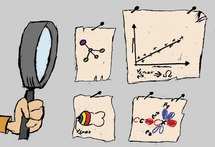

## Introduction

Halogen bonding denotes the non‐covalent interaction between electrophilic halogen substituents and Lewis bases.^1^ Complexes of this type have been known for more than 200 years.^2^ In the 1950s and 1960s their most important structural features have been elucidated^3^ and a theoretical model for such interactions based on charge transfer has been proposed by Mulliken.^4^ Still, there have been only a few studies in this area of research had been carried out before the mid‐1990s for the solid phase^5^ and before the mid‐2000s for complexes in solution.^6^ By now, however, halogen bonding is firmly established in crystal engineering^7^ and has found various applications in solution, including in anion binding,1c, 8 molecular recognition^9^ as well as organocatalysis.^10^

**Figure 1:**
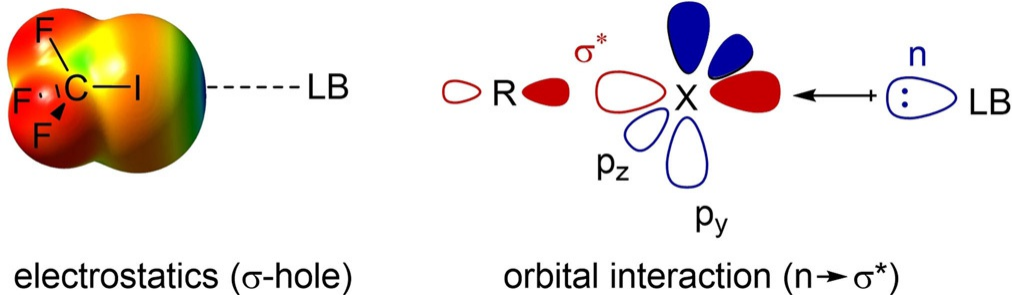
Halogen bonding: electrostatic (left) and orbital contributions (right). LB=Lewis base, X=halogen.

One possible reason for the low popularity of halogen bonding for most of the last century may be that it is counterintuitive based on a Lewis structure formalism: it is not immediately obvious why Lewis bases should form attractive interactions to halogen substituents, which themselves feature three electron lone pairs. In view of this, it is important to note that halogen bonding is not merely a van der Waals‐type attraction, but is based on multiple attractive electronic components. Next to the n→σ* charge‐transfer model proposed by Mulliken (Figure 1, right),4a a further popular rationalisation of halogen bonding is the electrostatic attraction between a region of positive charge on the halogen substituent and the Lewis base. The former has been termed the “σ‐hole”^11^ and plots of it have become very popular to demonstrate the Lewis acidity of suitable substituted halogens (Figure 1, left). This has led to claims that halogen bonding is purely electrostatic,^12^ and the Lewis acidity of halogen‐containing compounds is often, either explicitly or implicitly, assumed to be proportional to the “depth” of the σ‐hole (i.e., the most positive electrostatic potential on the surface of the halogen).11a, 13 On the other hand, various experimental^14^ and computational^15^ studies have clearly demonstrated the crucial role of charge transfer for halogen‐bonding complex formation and as a consequence, the relative importance of these two contributions to halogen‐bonding strength is currently under discussion.^16^

**Figure 2:**
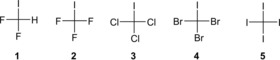
Difluoroiodo‐ and trihaloiodomethanes (**1**–**5**), increasing homologues from left to right.

**Figure 3:**
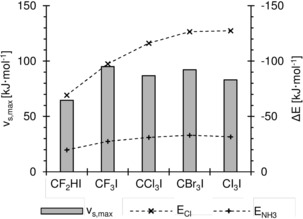
Calculated electronic association energies (*y* axis on right) of compounds **1**–**5** (*x* axis) with ammonia (+) and chloride (×) in comparison with their σ‐hole maxima (*v*_s,max_, *y* axis on left).

In the context of our studies to utilise halogen bonding in organocatalysis, we had performed orientating computations on simple halogen‐bond donors (halogen‐based Lewis acids) and had found an unexpected trend in the series of complexes CX_3_I⋅⋅⋅Cl^−^ and CX_3_I⋅⋅⋅NH_3_ (X=F, Cl, Br, I).^17^ For instance, the trifluoromethyl group is surely more electronegative than the triiodomethyl group, and thus CF_3_I (**2**) should feature a deeper σ‐hole than CI_4_ (CI_3_I, **5**) and should consequently also form stronger complexes to both Lewis bases (Figure 2). The opposite is the case, however, as has recently been re‐confirmed by high‐level calculations and an energy decomposition analysis.^18^ These studies15b, 18d, 19 point towards charge transfer (n→σ* orbital interactions) as the dominant factor in these adducts, which also over‐rides the trend predicted by pure electrostatics. So, although the depth of the σ‐hole (*v*_s,max_) seems to correlate very well with Lewis acidity for a range of halogen‐bond donors,^20^ there are clearly also limitations on its predictive value (Figure 3).

Herein, we aim to address this issue by searching for an overall better parameter to predict the halogen‐bonding strength of halogen‐bearing compounds.^21^ A variety of potentially suitable parameters (and their combinations) will be screened for correlation with halogen‐bonding adduct strengths. Considering the numerous indications on the relevance of charge transfer mentioned above, all parameters related to this electronic contribution are an obvious first alternative to σ‐hole depth. Ideally, a parameter for halogen‐bonding strength should possess the following properties:

- it should be conceptionally simple and easily obtainable by DFT calculations,
- it should be based on one calculation of the Lewis acid structure only (i.e., it should not require the computation of a complex, which would render this endeavour quite pointless, and ideally it also should not require the calculation of multiple states of the Lewis acid),
- it should be as general as possible,
- it should be susceptible to automation/scripting (i.e., it should not require selection of any parameter by the human eye).

Since solvent effects would very likely complicate the analysis dramatically, we will focus solely on gas‐phase interactions.^22^ The reliability and quality of the benchmarking results will obviously strongly depend on the quality of the interaction energies, against which all parameters will be tested. Thus, an important first step will be the generation of high‐quality coupled‐cluster energies and a validation of DFT energies against this standard.

## Results and Discussion

### Benchmarking of DFT^23^ methods against CCSD(T)^24^

Since it is not feasible, especially for larger systems, to compute CCSD(T) CBS data in order to assess the Lewis acidity of halogen‐bond donors, our first goal was to find a DFT method that would produce comparable binding data at much lower costs. We started with some common DFT functionals, some of which have already performed well in benchmarking by Grimme and co‐workers.^25^ Herein, we used the B3LYP,^26^ B97D3,^27^ M05‐2X,^28^ M06‐2X,^29^ mPW1PW91^30^ and ωB97xD^31^ functionals, with and without Grimme dispersion correction,^32^ in combination with the Karlsruhe basis sets.^33^ Triple‐ζ basis sets were mostly applied with one or two polarisation functions. In addition, the halogens chlorine to iodine were augmented with a diffuse function derived by Rappoport and Furche^34^ (this basis set is denoted as def2‐TZVP(D)^22^, ^35^) based on the well‐known issues of DFT with anions and our own experience of solution‐phase calculations.^22^, ^36^

**Figure 4:**
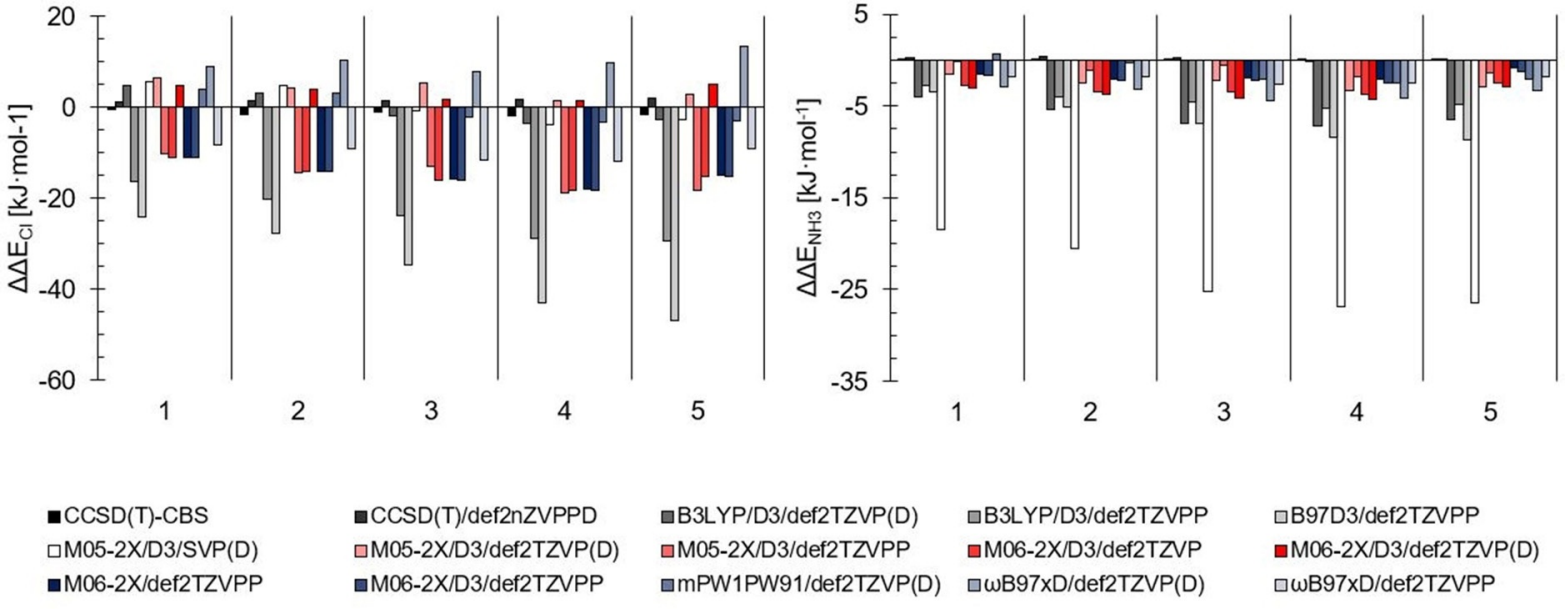
Difference of the extrapolated CCSD(T)/aug‐cc‐pVnZ(‐PP) association energies of the halomethanes **1**–**5** with chloride (left) and ammonia (right), and the results of thirteen DFT functional basis set combinations, as well as two more extrapolated coupled cluster energies. The DFT results are grouped according to the compound **1**–**5**, and each bar comprises the energy difference for one of the functionals. Overall M05‐2X/D3, M06‐2X/D3 and mPW1PW91 (all with the def2‐TZVP(D) basis set) show the lowest difference in association energies compared with the coupled cluster results. M05‐2X/D3/def2‐SVP(D) shows outstanding performance of the energies with chloride, but delivers the worst results for ammonia.

In our previous study,18d ΔCCSD(T) def2‐QZVPPD data were obtained, with two‐point complete basis set limit extrapolated MP2 energies,^37^ for the complexes of halomethanes **2**–**4** (Figure 2) with chloride and ammonia. Herein, we also included difluoroiodomethane (**1**) and applied the extrapolation method of Feller as given by Vasilyev^38^ on the aug‐cc‐pVnZ (n=2–4) basis set and the procedure of Halkier et al. for the extrapolation of the MP2 energies on the def2‐nZVPPD and aug‐cc‐pVnZ‐(PP) (n=3–4) basis sets.^39^ As expected, the difference between the chosen methods is only in the range of 4 kJ mol^−1^ (Figure 4). These further options are particularly important for larger molecules, which become impossible to compute with fully augmented Dunning basis sets.

Earlier computational benchmark studies on halogen bonding were conducted by Řezáč et al.^40^ and Kozuch and Martin,15c who employed their XB16, X40 and XB51 sets of halogenated molecules, which describe complexes of uncharged interacting partners. Herein, we focus mostly on C−I‐based halogen‐bond donors and their interactions with chloride and ammonia as prototypical anionic and neutral Lewis bases.

Figure 4 also includes the DFT results of the computed association energies between halogen‐bond donors **1**–**5** and chloride or ammonia.

For the ammonia complexes, most functional/basis set combinations perform reasonably well once triple‐zeta basis sets and dispersion corrections are used. Under these conditions, B3LYP and B97D3 show the largest errors, whereas the other functionals (M05‐2X, M06‐2X, mPW1PW91 and ωB97xD) yield very good results. With regard to basis sets, def2‐TZVPP naturally outperforms def2‐TZVP in accuracy, but even for these comparably small complexes, calculations take markedly more time.

For the complexes involving chloride as Lewis base, M05‐2X/D3/SVP(D) performs surprisingly well, particularly in comparison with the TZVP variants. Because this is likely a fortunate cancellation of errors, it was not considered further, also in the light of the very bad results obtained for the ammonia complexes. For the triple‐zeta basis sets, overall, the errors are larger compared with the neutral complexes. B3LYP and B97D3 once again provide the largest deviations, whereas the Minnesota functionals with the def2‐TZVP(D) basis set generate very low errors. Overall, M05‐2X/D3/TZVP(D) reproduces the benchmark values best and was thus (pre)selected as the functional of choice.^41^ These findings seem to be in agreement with the results of Kozuch and Martin,15c who recommend the application of the M06‐2X and ωB97xD functionals, which also performed well in our case. In their study, the related M05‐2X functional was not tested, and because no anions were included, no diffuse functions were added to the def2‐TZVP/PP basis set.

**Figure 5:**
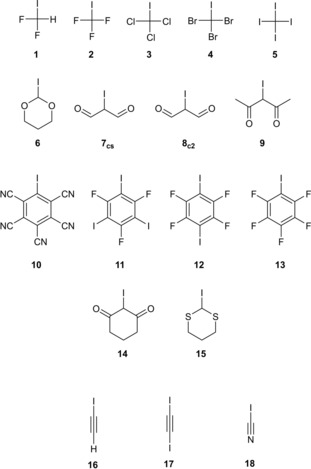
Overview of the test set molecules **1**–**18**, including five halomethanes **1**–**5**, six more sp^3^‐hybridised XB donors featuring more or less electron‐withdrawing oxygen and sulfur substituents (**6**–**9**, **14** and **15**), four aromatic systems, three of them polyfluorinated (**11**–**13**) and the very electron‐deficient pentacyanoiodobenzene (**10**), and three alkyne‐bonded iodine compounds (**16**–**18**).

Next, we extended our set of halogen‐bond donors to see whether M05‐2X/D3/TZVP(D) would also reproduce the CCSD(T) CBS data for a more diverse array of compounds (Figure 5). As most applications of halogen bonding are based on iodine‐bearing carbon backbones,10e we focused on halogen‐bond donors featuring C−I bonds and strived to select representative small‐molecule examples for all variations of hybridisation with different electron‐withdrawing substituents. Thus, six sp^3^‐hybridised compounds **6**–**9**, **14** and **15** were additionally included, which feature α‐carbonyl substituents or (thio)acetal moieties as polarising groups. In addition, four aromatic compounds **10**–**13** were selected, three of which represent very common halogen‐bond donors with polyfluoro/polyiodo substituents. In the fourth compound, nitrile groups replace the electron‐withdrawing fluorine atoms because Grimme and Matzger and co‐workers have shown that even stronger halogen‐bond donors can be obtained with these substituents.^44^ Finally, three examples of sp‐hybridised alkynes, **16**–**18**, were also included in the study. These molecules will be called the test set from here on.

**Figure 6:**
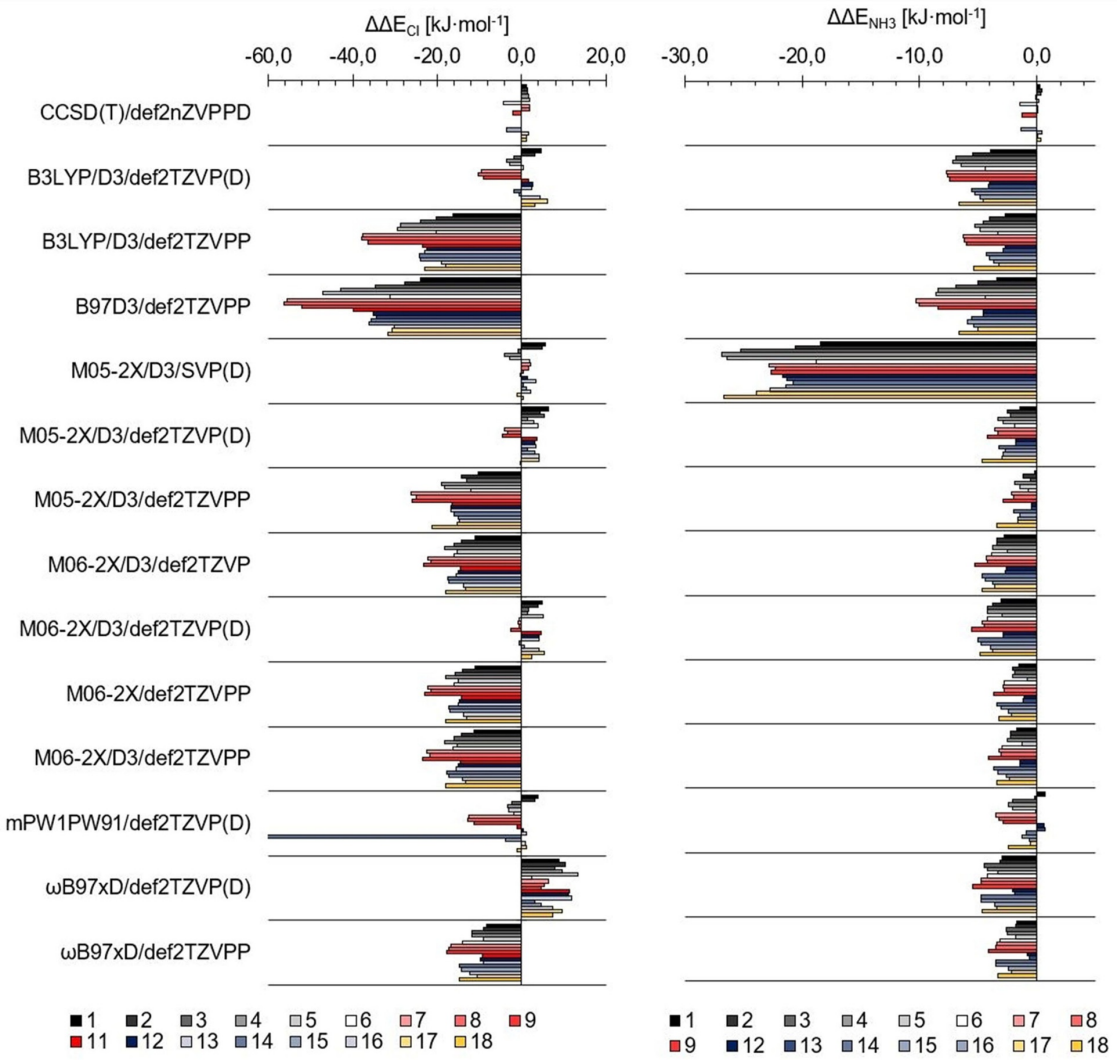
Difference of the extrapolated CCSD(T)/(aug)‐cc‐pVnZ(‐PP)^42^ association energies of the test set with chloride (left) and ammonia (right), and the results of 13 DFT functional basis set combinations, and the extrapolated results for CCSD(T)/def2‐nZVP. In contrast to the previous plot, the results are grouped according to the applied basis set (and cc), in alphabetical order. The energy differences for each compound **1**–**18** are shown as coloured bars.^43^ The overall trend observed for the halomethanes is the same and M05‐2X/D3/def2‐TZVP(D) produces the lowest overall error (compare with the sum of absolute errors in Table 2).

**Figure 7:**
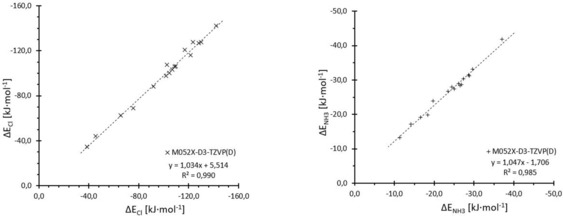
Direct comparison of the association energies from the extrapolated coupled cluster results (*x* axes) for the test set with the energies from M05‐2X/D3/def2‐TZVP(D) (*y* axes) with chloride (×, left) and ammonia (+, right). Both sets of data show a high linearity with slopes close to 1 and *R* ^2^ values of 0.99. The intercept for ammonia is at −1.7 kJ mol^−1^, with M05‐2X underestimating the binding energy slightly, whereas the binding energy of chloride with an intercept of 5.5 kJ mol^−1^ is overestimated.

We again compared the binding energies of the test set molecules to chloride and ammonia, as obtained by our chosen functionals, with those of the extrapolated CCSD(T) energies (Table 1), as shown in Figure 6 and Table 2, and found a satisfactory agreement for M05‐2X and M06‐2X with D3 correction by using the def2‐TZVP(D) basis set and mPW1PW91/def2‐TZVP(D). Figure 7 additionally shows the excellent correlation of the M05‐2X results with the coupled cluster data.

**Table 1:** Extrapolated39c CCSD(T)/aug‐cc‐pVnZ(‐PP) electronic binding energies for complexes **1**–**18** with chloride and ammonia.

	Δ*E* [kJ mol^−1^]	
	Cl^−^	NH_3_
**1**	−75.58	−18.35
**2**	−101.88	−24.94
**3**	−121.51	−28.85
**4**	−127.96	−29.71
**5**	−130.29	−28.66
**6** ^[a]^	−38.62	−11.43
**7** ^[a]^	−123.31	−24.44
**8** ^[a]^	−117.24	−23.37
**9** ^[a]^	−102.87	−19.79
**10** ^[a]^	–^[b]^	–^[b]^
**11** ^[a]^	−106.83	–^[b]^
**12** ^[a]^	−109.37	−26.59
**13** ^[a]^	−109.14	−26.86
**14** ^[a]^	−45.50	−14.07
**15** ^[a]^	−65.81	−16.52
**16** ^[a]^	−92.05	−26.14
**17**	−104.36	−27.44
**18**	−142.08	−37.06

[a] Instead of using aug‐cc‐PVnZ(‐PP) on all atoms, only Cl and I were augmented. [b] The calculation of the triples did not converge.

**Table 2:** Comparison of the absolute errors for each tested functional basis set combination over the full test set (**1**–**18**). The lowest errors are highlighted in blue, medium in white and the highest errors in red.

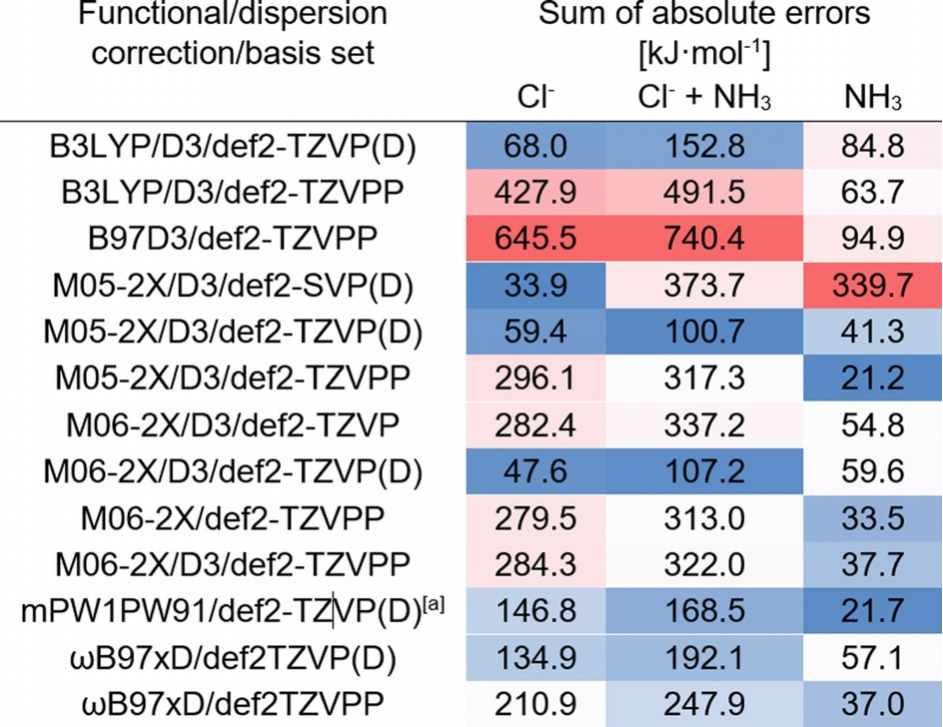

[a] mPW1PW91 gives a different minimum for one of the compounds, otherwise it is as good as M05‐2X.

### Evaluation of potential parameters for halogen‐bonding strength in the test set

**Figure 8:**
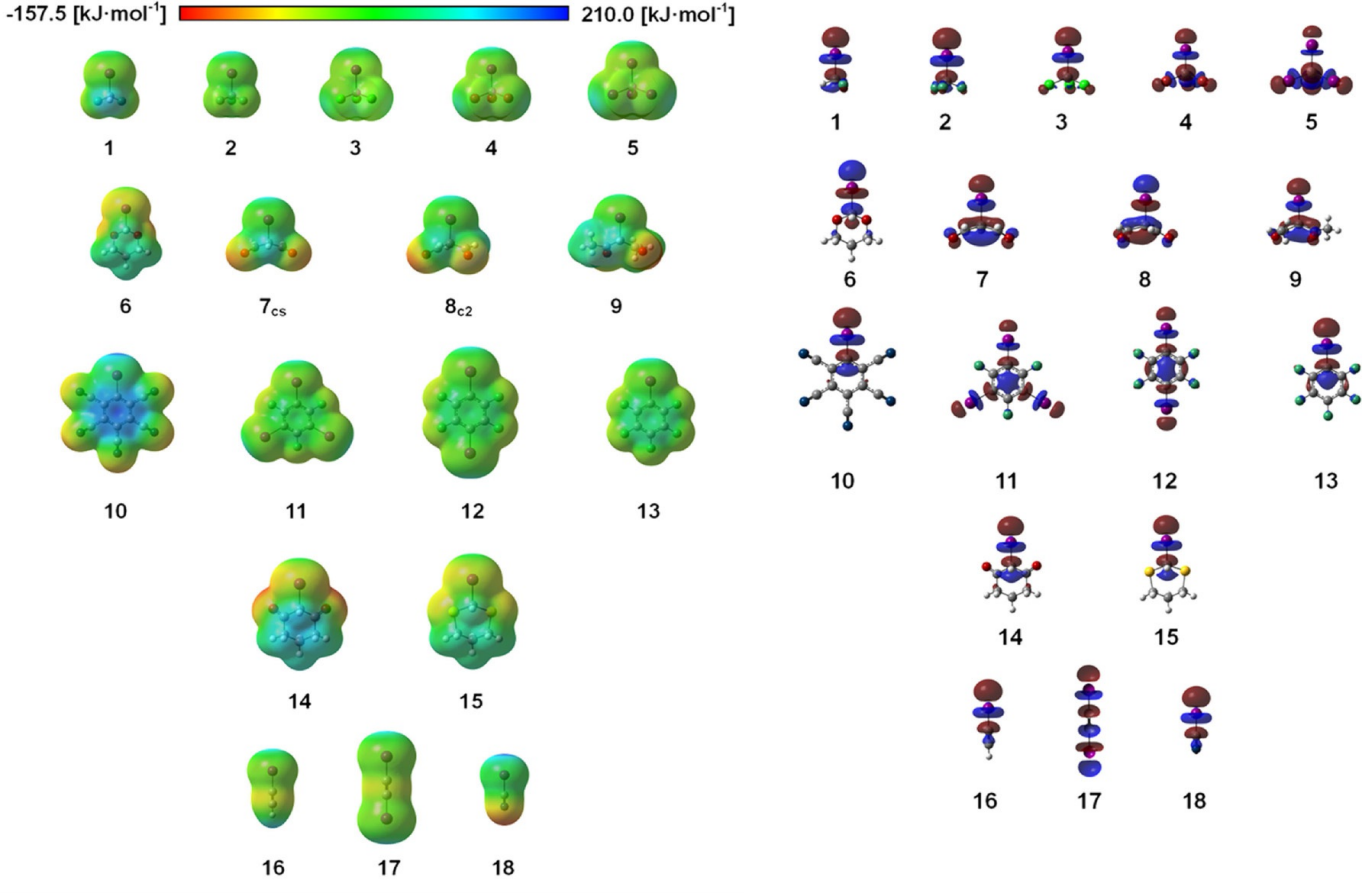
Electrostatic potentials for the test set compounds **1**–**18** computed at the 0.001 electron Bohr^−3^ isodensity surface (left) and the σ* orbitals (right). All molecules are oriented as in Figure 1 so that the iodine atoms, σ‐holes and σ* orbitals of interest point in the same direction and *v*_s,max_ is always at the tip of each compound. The scale (−157.5 to 210.0 kJ mol^−1^) was chosen in such a manner that the whole potential range is visible. Note the difference in electron density of the aromatic rings in compounds **10** and **13**. A further graphic showing all computed electrostatic potentials in this paper from a slightly different perspective (to better visualise the σ‐hole) is provided in the Supporting Information.

Having established the feasibility of using DFT energies to study halogen‐bonding strength, we next turned our attention towards individual parameters of the halogen‐bond donors that might allow prediction a priori of their halogen‐based Lewis acidity. The obvious first choice for such a parameter is the depth of the σ‐hole of each compound, that is, the most positive electrostatic potential on the surface of the iodine substituents (which usually occurs at an angle of 180° relative to the C−I bond). Figure 8 (left) shows the electrostatic potential maps of all compounds **1**–**18**.

**Figure 9:**
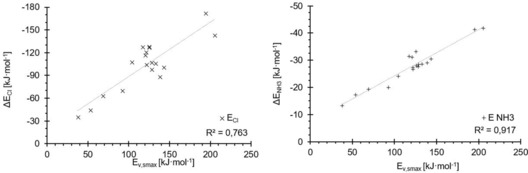
Electrostatic potentials (*x* axes) were computed at the 0.001 electron Bohr^−3^ isodensity surface and the resulting σ‐hole maxima (*E*_vs,max_) were plotted against the binding energies of the complexes derived from M05‐2X/D3/def2‐TZVP(D) (*y* axes). Linear correlation coefficients: 0.76 (chloride (×), left) and 0.92 (ammonia (+), right). Although the plot for the ammonia complexes follows a general order, with only three outliers, the same plot for chloride shows a significant amount of disorganisation in the range between −88 and −130 kJ mol^−1^_,_ which is the region in which most of the halomethanes and aromatic compounds are located.

Plots of the depth of the σ‐hole of each test set molecule versus its M05‐2X/D3/def2TZVP(D) binding energy to chloride (Figure 9, left) or ammonia (right) reveal a relatively decent correlation for the latter Lewis base (*R* ^2^=0.92), but a rather modest one for the chloride complexes (*R* ^2^=0.76). The correlation of σ‐hole depth with the halide binding energies would be even worse if the strongest‐ and weakest‐bound complexes were omitted and only the halogen‐bond donors with σ‐hole energies between −69 and −143 kJ mol^−1^ were considered. In this region, which comprises the majority of test set molecules, there is virtually no trend at all between the two parameters. In the ammonia complexes, the correlation is much better in this region of σ‐hole depth and the overall trend is followed. The two strongest halogen‐bond donors, ICN and C_6_(CN)_5_I, feature very similar σ‐hole depths and their binding to ammonia is also virtually identical in strength (with a difference of less than 1 kJ mol^−1^). In contrast, the complexation energies with chloride differ in stability by more than 30 kJ mol^−1^.

Chloride as a stronger Lewis base than ammonia will lead to halogen‐bonded complexes in which charge transfer is more relevant (and electrostatics are less relevant) than for neutral adducts. This was recently confirmed for the interactions of CX_3_I (X=F to I) with both substrates.^45^ Thus, it is not surprising that the interactions with the halide are modelled less well by the (static)^16^ σ‐hole energies, that is, an electrostatic approach. As a consequence, we turned our attention towards the orbitals of the Lewis acids, which seemed relevant for charge transfer, and evaluated whether their energies would lead to a better correlation with Lewis acidity, either for the entire test set or at least for a subset. The orbitals considered were the LUMO of each molecule, the molecular orbital that seemed to best represent the C−I σ* orbital (after visual inspection of all low‐energy MOs), denoted σ*_MO_, and the σ* orbital according to a natural bond order (NBO) analysis (called σ*_NBO_).^46^

**Figure 10:**
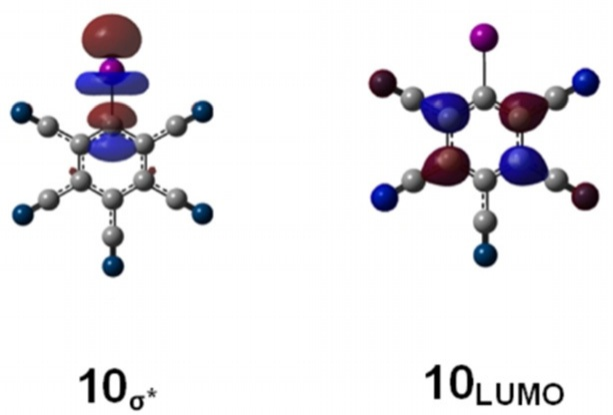
Comparison of the σ*_MO_ orbital (top) and the LUMO (bottom) of pentacyanoiodobenzene (**10**).

**Figure 11:**
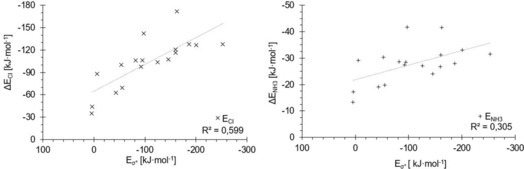
Halogen‐bonding energies of the test set molecules (*y* axes) with chloride (×, left) and ammonia (+, right) versus σ*_MO_ orbital energies (*x* axes). Both plots show far worse correlations than the *v*_s,max_ energies, although in the region of intermediate binding energies to chloride (−88 to −130 kJ mol^−1^), the order of compounds is reproduced better.

**Figure 12:**
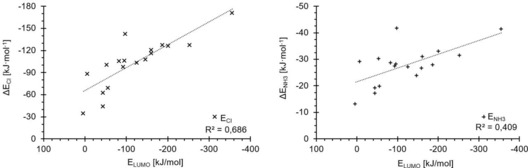
LUMO energies (*x* axes) versus halogen‐bonding energies (*y* axes) for the chloride (×, left) and ammonia (+, right) complexes of the test set molecules.

For the vast majority of the compounds in the test set, the LUMO and σ*_MO_ are identical, with very few exceptions, like C_6_(CN)_5_I (Figure 10). Thus, plots of the complexation energies versus the orbital levels are naturally very similar for both the σ*_MO_ and LUMO (Figures 11 and 12).

For σ*_MO_, both correlation coefficients are much lower than for the σ‐hole plots (0.60 for the chloride and 0.31 for the ammonia complexes). For ammonia, in particular, there is little predictive value of these orbital levels. A visual (and entirely subjective) inspection of the chloride‐binding correlation, however, seems to indicate that the overall trend is reproduced reasonably well, but is spoiled by several outliers. In any case, despite the overall weaker correlations compared with the σ‐hole parameter, it is noteworthy that in contrast to the latter, σ*_MO_ provides a better prediction of the chloride complexes than the ammonia complexes. This is again in line with a more important contribution of charge transfer in the charge‐assisted adducts.

As indicated above, comparable results were obtained with the LUMO energies as parameter (for plots see Figure 12). The correlation coefficient for the chloride complexes is virtually identical (*R* ^2^=0.69), whereas the one for the adducts involving ammonia is somewhat better, but overall still quite poor (*R* ^2^=0.41). A significant contribution to the slightly better fit of the neutral complexes stems from the strong halogen‐bond donor C_6_(CN)_5_I. Although its LUMO is based on the π system of the aromatic core (Figure 10) and is thus not directly related to halogen bonding, its energy level fortuitously provides a better estimation of the interaction energy.

**Figure 13:**
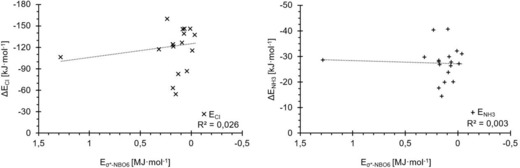
Energies computed for the σ* orbitals using NBO analysis (*x* axes) show no linear correlation with the binding energies (*y* axes) for the chloride (×) and ammonia (+) complexes even without the outlier at about 1.3 MJ mol^−1^.

Because the correlation coefficients for both σ*_MO_ and LUMO are relatively low, we also evaluated the σ* energies of the test set molecules as obtained by NBO 6.0^46^ analyses (σ*_NBO_). Somewhat surprisingly, there is no correlation at all between these energy levels and the corresponding complex stabilities, neither for chloride nor for ammonia (see Figure 13). For similar values of σ*_NBO_ energy, a wide range of possible complex energies is obtained, with no apparent trend. In addition, one σ*_NBO_ energy level (at 1.3 MJ mol^−1^, corresponding to **17**) strongly deviates from all the others, which was not observed for the σ*_MO_ and LUMO orbitals.

Overall, none of the orbital levels considered (σ*_MO_, LUMO or σ*_NBO_) provides a correlation that is competitive with that of the σ‐hole depth. In contrast to the latter, the stronger charge‐assisted complexes are described better than the neutral ones, but as a single parameter, the predictive value is very limited. Of the three alternatives, the LUMO levels seem to be the best option as they do not involve the possibly arbitrary selection of a “most σ*‐like” orbital by a human, which would also not be susceptible to scripting/automation (even though the nature of the LUMO orbital may not intuitively be connected to the n→σ* description of halogen bonding).

Despite the disappointing performance of the σ*_NBO_ levels, several other parameters that could be obtained by an NBO analysis, and that seemed more or less directly related to halogen bonding, were also considered as potential indicators. These include the charge on the iodine substituent (*Q*_I_), the charge on the iodine‐bearing carbon atom (*Q*_C_), the hybridisation of the latter (Csp^*X*^) and the percentage of the σ* orbital attributed to iodine (%σ*).

None of these parameters yielded satisfactory results: the *Q*_C_ data set show no apparent correlation (see Tab M06‐2X in the Supporting Information), and although the *Q*_I_ plots at least provide discernible trend lines, their correlation coefficients (*R* ^2^=0.32 for chloride and *R* ^2^=0.56 for ammonia) are still quite lacking. Even worse correlation coefficients were obtained for Csp^*X*^ and %σ*. The latter yielded a slightly better linear trend than either *Q*_C_ or Csp^*X*^, but a worse one than *Q*_I_. Interestingly, all of these parameters described the ammonia complexes somewhat better than the chloride adducts, which is somewhat unexpected given the more electrostatic nature of the former.

A final potential indicator of Lewis acidity that can be directly obtained from the calculations of the halogen‐bond donors alone is the C−I bond length (*d*_C−I_). The corresponding plots versus the binding energies (see Table M062X in the Supporting Information) clearly show the sets of compounds with differently hybridised carbon atoms, but fail to demonstrate any clear trend. For halogen‐bond donors with the same hybridisation of the C−I carbon, very similar C−I lengths are obtained, whereas the interaction energies of these Lewis acids differ by more than 50 kJ mol^−1^ for chloride and more than 10 kJ mol^−1^ for ammonia.^47^

**Figure 14:**
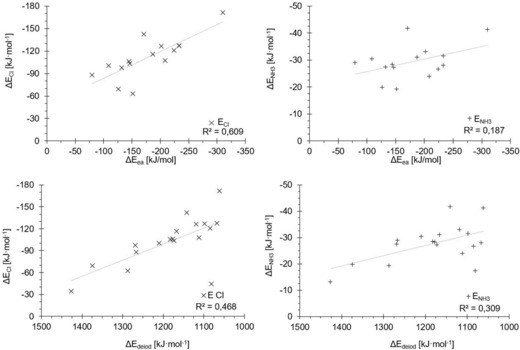
Electron affinities Δ*E*_ea_ (top) and deiodination energies Δ*E*_deiod_ (bottom) for the test set, each versus the association energies with chloride (×, left) and ammonia (+, right). Due to unwanted deiodination processes during the calculations, only 14 of the 18 possible data points are featured in the electron affinity graphs.

Because none of the additionally tested parameters provide any improvement on the correlation obtained by the σ‐hole depth, we finally also considered data that require the computation of an additional species. As mentioned above, halogen bonding may be described as an electron donation by the Lewis base into the C−X σ* orbital of the halogenated compound. In the extreme case, this would lead to dehalogenation (here, deiodination) and the formation of the corresponding carbanion after transfer of the iodonium cation (I^+^) to the substrate. To model the partial electron transfer, the electron affinities of the halogen‐bond donors were estimated by subtracting the energies of the corresponding radical anions from those of the neutral Lewis acids. This approach was not without its problems, as for example, the radical anion of **6**, among others, deiodinated during the geometry optimisation, liberating a hydrogen‐bonded iodide. Plots of this estimated electron affinity (*E*_ea_) versus complex stabilisation resulted in a mediocre correlation for chloride (*R* ^2^=0.61) and a very weak one for ammonia (*R* ^2^=0.19) (Figure 14, top). Because this does not even improve the performance of the LUMO energies, but requires an additional calculation per Lewis acid, it was not further considered.

An alternative approach is to model the partial deiodination by subtracting the energies of the respective carbanions from those of the halogen‐bond donors. In contrast to the radical anions, all the carbanions could be obtained without difficulty. The trend lines of the deiodination energy (*E*_deiod_) versus the binding energies, however, provide even worse correlation coefficients than those for the radical anions (*R* ^2^=0.47 for chloride and *R* ^2^=0.31 for ammonia, Figure 14, bottom).

To summarise the findings of this section, the best single parameter to predict the Lewis acidity is the σ‐hole depth, even though it is less than ideal for the charge‐assisted chloride complexes and also fails to put simple halogenated compounds like CX_3_I (X=F to I) in the correct order.18d, 45 Second best are the LUMO and σ*_MO_ energy levels, which provide somewhat acceptable correlations for the halide complexes but poorly describe the ammonia ones. None of the other parameters considered provided an improved fit, even when electron‐transfer processes were modelled by additional calculations.

### Linear combinations of two or more parameters

Since no other single parameter could remedy the shortcomings of the σ‐hole approach, we wondered whether a linear combination of two (or more) parameters would provide a superior descriptor. Thus, we defined a new parameter, *Ω*, which combines the σ‐hole depth with one (or more) other parameter(s), all expressed in kJ mol^−1^, according to Equation (1).

(1) $$\Omega =aE{}_{\sigma \;\mathrm{hole}}+bE{}_{\mathrm{Parameter}1}\left(+cE{}_{\mathrm{Parameter}2}\right)$$

We stress that this is a purely empirical approach in which the coefficients *a* and *b* (and possibly *c*) are optimised iteratively until the best correlation is obtained. During optimisation, a further arbitrary condition was that *Ω* of the strongest halogen‐bond donor, C_6_(CN)_5_I, should be less than one to limit the range of *Ω* to values between 0.0 and 1.0.

Intuitively, it seems that the most promising candidate for the second parameter would be one that relates to “charge transfer”,^16^ to counterbalance the shortcomings of the σ‐hole depth, a purely electrostatic descriptor, for the stronger complexes. Because we have seen above that the LUMO or the σ*_MO_ energies constitute the second‐best parameter, we first tested the combination of σ‐hole depth with σ*_MO_ energies.

**Figure 15:**
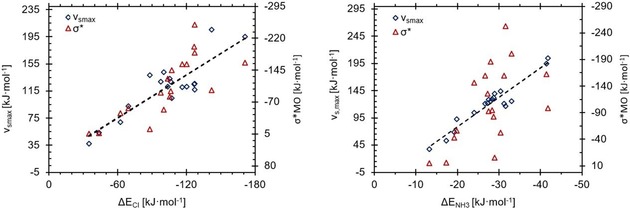
Energies of the σ‐holes (*y* axes on left) and σ* orbitals (*y* axes on right) plotted against the binding energies (*x* axes, Cl^−^ on the left and NH_3_ on the right) in such a manner that the linear regression curves overlap.

Prior to the actual optimisation of the coefficients, we first prepared an overlay of the correlations of both σ‐hole depth and σ*_MO_ versus the binding energy in such a fashion that the latter values are on the *x* axis, and the two separate *y* axes represent the values of both parameters (Figure 15). The scale was adjusted so that the correlation trend lines would overlap. This was meant to provide a quick qualitative impression of the feasibility of the approach. Indeed, it seems that in most situations when one parameter strongly deviates from the trend line, the other parameter deviates in the opposite direction, so that a linear combination of the two would likely improve the overall fit.

**Figure 16:**
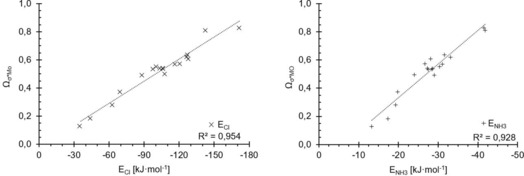
Plots of *Ω*_σ*MO_ (as the optimised linear combination of σ‐hole depth and σ*_MO_ energies, *y* axes) against the association energies (*x* axes) for chloride (×, left) and ammonia (+, right).

**Figure 17:**
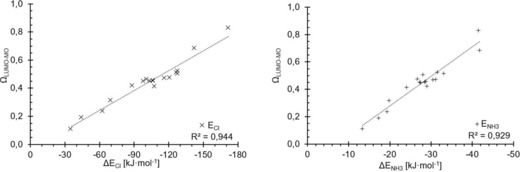
Plots of *Ω*_LUMO_ (as the optimised linear combination of σ‐hole depth and LUMO_MO_ energies, *y* axes) against complex binding energies for chloride (left) and ammonia (right).

The coefficients *a* and *b* were then optimised to provide the best correlation, and the resulting plots of *Ω*_σ*MO_ (with *a*=3.513×10^−3^ and b=−8.962×10^−4^) versus the binding energy are shown in Figure 16. For the ammonia complexes, the correlation coefficient is virtually identical to the one of the pure σ‐hole parameter (*R* ^2^=0.92/0.93), even though the position of the trend line has moved (compare Figure 5). A marked difference is observed, however, for the chloride complexes: the correlation is now excellent (*R* ^2^=0.95), especially in comparison with the results of the individual parameters (*R* ^2^=0.60 for σ*_MO_ and *R* ^2^=0.76 for σ‐hole depth). Thus, the combined parameter *Ω*_σ*MO_ now describes both prototypical binding situations very well, and does markedly better with the stronger, charge‐assisted cases, in contrast to the pure σ‐hole depth. With very few exceptions, the σ*_MO_ orbital also represents the LUMO of the respective molecule. Thus, the parameter *Ω*_LUMO_, which uses the LUMO energy regardless of the nature of the orbital (and is thus suitable for automatisation/scripting), provides correlations (Figure 17) that are almost as good as the ones of the “ideal” parameter *Ω*_σ*MO_ (Figure 16).

**Figure 18:**
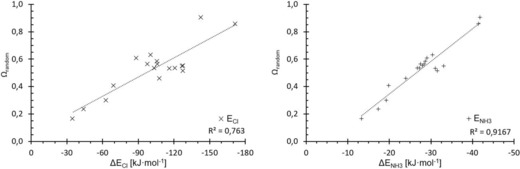
Plots of the linear‐combination parameter *Ω*_random_ (consisting of the σ‐hole depths and a random value for every compound of the test set) against the complex binding energies for chloride (left) and ammonia (right).

Naturally, linear combinations of other parameters with σ‐hole depth were tested as well, but none provided superior correlation. To rule out that the improvement in correlation is merely due to a statistical effect, we also tested a linear combination of σ‐hole depth with a randomly generated value for each halogen‐bond donor, *Ω*_random_. During the optimisation of the linear combination, the *b* coefficient converges to zero, however obviously resulting in virtually identical results to those obtained with the pure σ‐hole depth (Figure 18). Thus, the improvement in correlation seen above does not seem to be a mathematical artefact.

**Figure 19:**
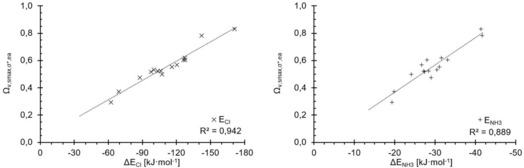
Performance of *Ω* resulting from the combination of three parameters: σ‐hole, σ*_MO_ and electron affinity.

Despite a strong motivation to keep the new descriptor as simple as possible, combinations of three parameters were also tested, most notably the one including σ‐hole depth, σ*_MO_ level and electron affinity *E*_ea_, three of the best individual parameters tested above. For the ammonia complexes, only a marginal improvement was observed and it seems that the correlation is already a maximum with two parameters (Figure 19, right). The very slight increase in the correlation coefficient for the chloride complexes (*R* ^2^=0.96) does not justify the additional effort to compute the electron affinities and thus it seems that no significant further improvement can be achieved with further parameters.

**Figure 20:**
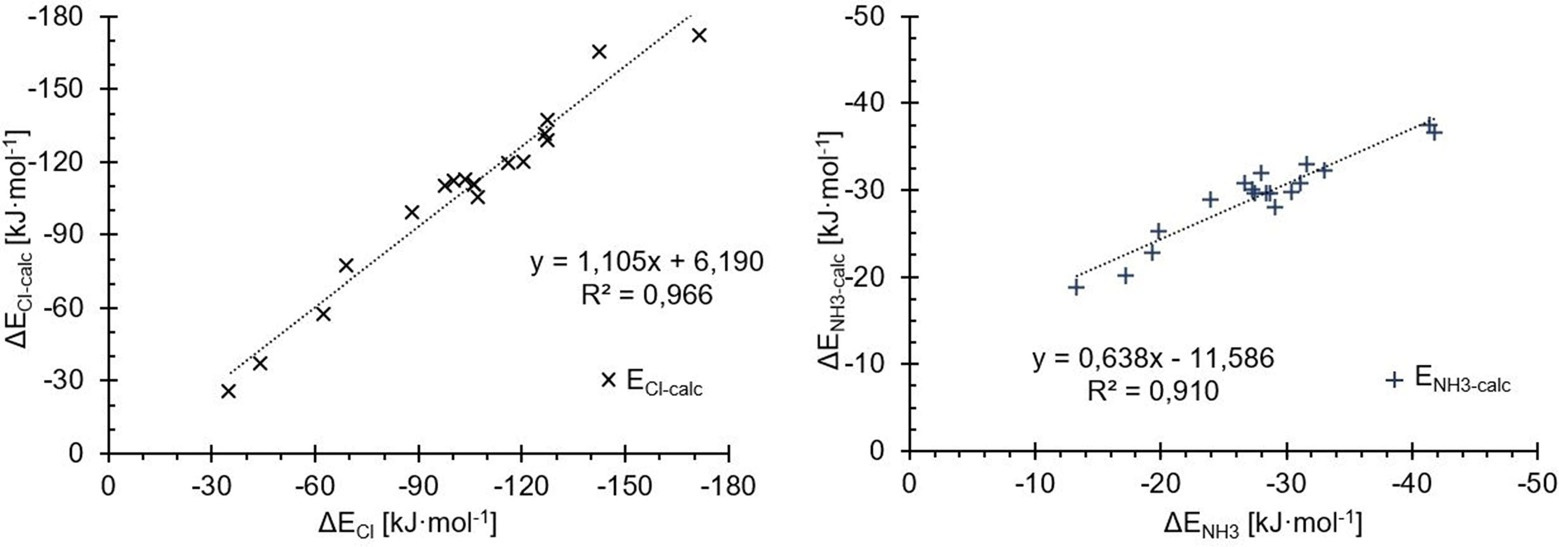
Electronic binding energies derived from gas‐phase computations using M05‐2X/D3/def2‐TZVP(D) versus the energies calculated with *Ω*_σ*MO_ for chloride (left) and ammonia (right).

Finally, to visualise the predictive value of the best descriptor *Ω*_σ*MO_, the interaction energies of all the chloride and ammonia complexes were calculated by multiplication of *Ω*_σ*MO_ with an appropriate constant [Eqs. (2), (3)], and these binding energies were plotted against the corresponding values as obtained by DFT (Figure 20). Both trend lines intercept close to zero, with slopes close to 1 (see Figure 20 for details) and hardly any outliers.

(2) $$E_{{CI}^{-}}=-182.45\Omega {}_{\sigma *\mathrm{MO}}-8.5$$

(3) $$E_{{NH}_{3}}=-38.84\Omega {}_{\sigma *\mathrm{MO}}-7.61$$

 

### Validation

**Figure 21:**
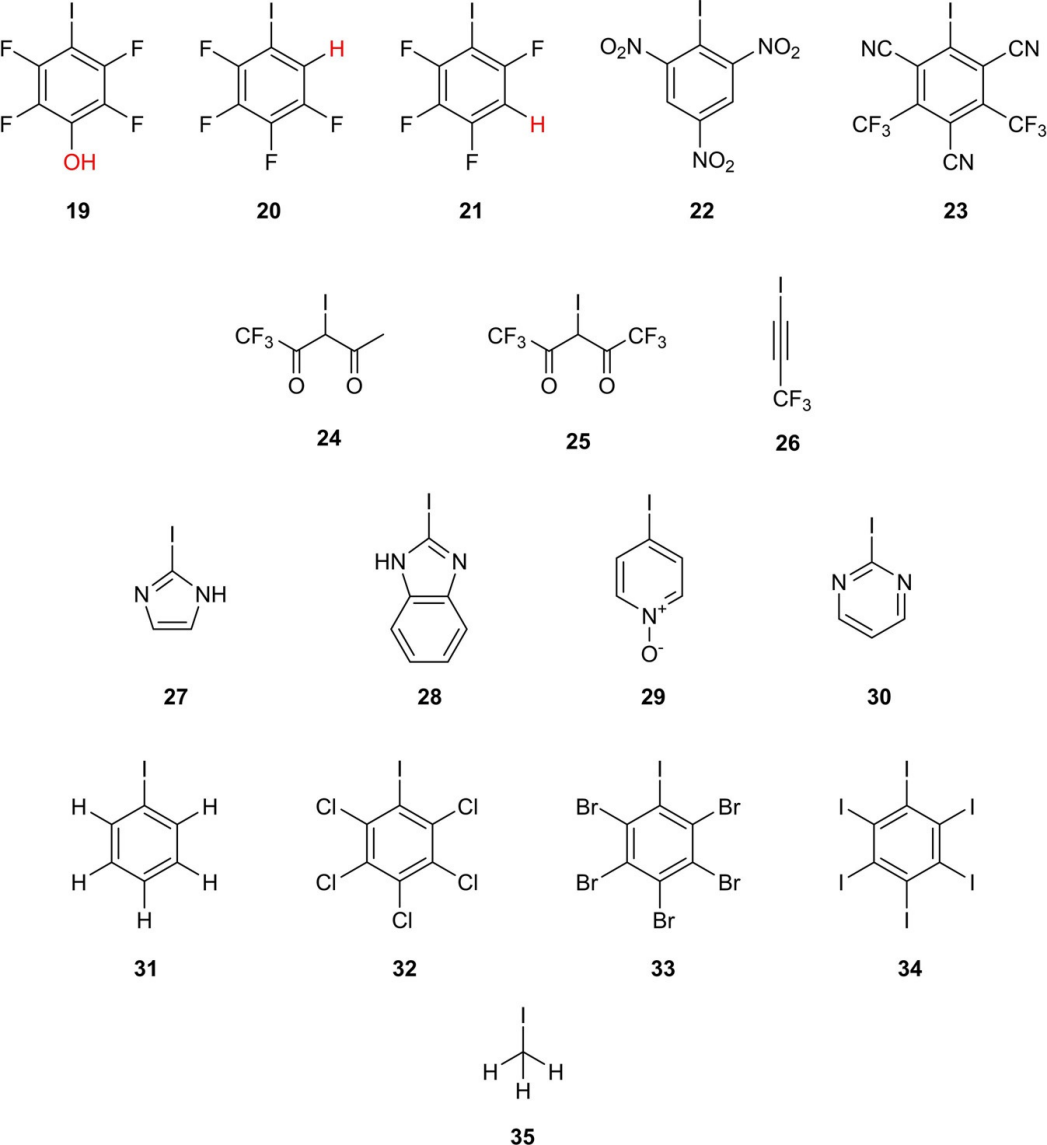
Compounds **19**–**35** of the validation set (val) featuring 13 aromatic compounds, two C(sp^3^)−I‐based compounds and one C(sp)−I‐based compound, with iodomethane (**35**) and iodobenzene (**31**) as examples of very weak Lewis acids.

Equipped with an improved predictor of halogen‐bonding‐based Lewis acidity, *Ω*_σ*MO_, we next tested its performance with halogenated compounds that were not part of the test set in the benchmarking process. This additional group of molecules, called the validation set from here on, includes halogen‐bond donors that were expected to bind more weakly as well as more strongly than the ones in the test set to explore the limits of our empirical approach. The 17 compounds, **19**–**35**, selected are depicted in Figure 21. Three Lewis acids featuring an sp^3^‐hybridised carbon centre were chosen, with iodomethane **35** as a very weak halogen‐bond donor and compounds **24** and **25** as more Lewis acidic derivatives of dicarbonyl **8** of the test set. In addition, nine aromatic compounds with various additional substituents next to the iodine were also included. These range from iodobenzene (**31**), a weak Lewis acid, to derivatives **22** and **23**, which feature strongly electron‐withdrawing nitro, trifluoromethyl and cyano groups. Four heteroaromatic compounds, **27**–**30** (including the pyridine *N*‐oxide **29**), constitute a class of halogen‐bond donors that was not part of the test set and should thus provide a challenge to Lewis acidity prediction. Finally, an electron‐deficient derivative, **26**, of the iodoalkynes **16** and **17** in the test set was included.

**Figure 22:**
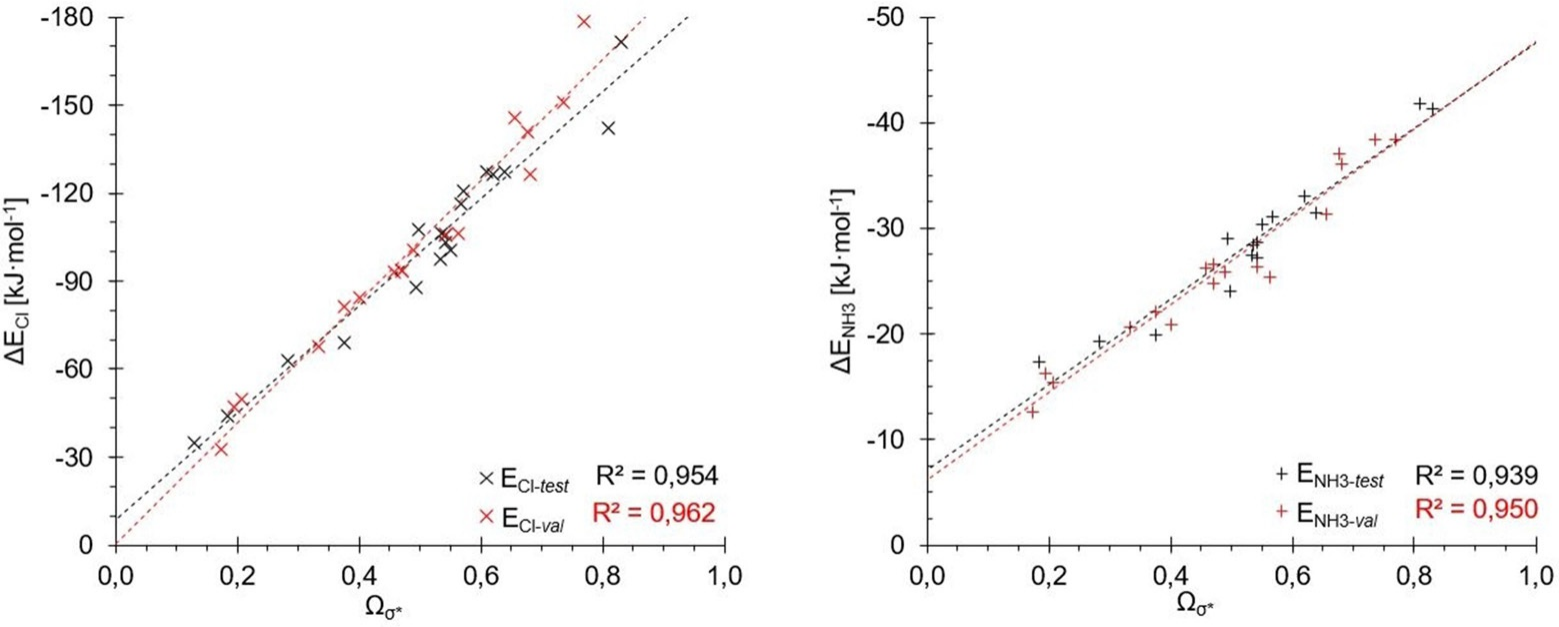
Comparison of the *Ω*_σ*_ parameter for the test (black) and validation set (red) compounds with the association energies with chloride (×) and ammonia (+). All four determined *R* ^2^ values are around 0.95, with the slopes and intercepts of the ammonia lines being almost identical and a small difference for the chloride curves, possibly due to the excess of aromatic compounds in the validation set compared with in the test set.

For each validation set (val) molecule, its *Ω*_σ*_ parameter as well as its binding energies to chloride and ammonia were calculated with M05‐2X/D3/def2‐TZVP(D). The resulting correlations (in red) are compared with the ones of the test set (in black) in Figure 22. For the ammonia complexes, the correlation of the validation set is again excellent (*R* ^2^=0.95) and the two trend lines are virtually identical, despite the structurally much more diverse set of molecules in the validation set. For the chloride complexes, the correlation coefficient (*R* ^2^=0.96) and the fit are also very good. However, the slope of the trend line is slightly different, which would lead to a deviation in the prediction of the absolute binding energies, but these would still show reasonable accuracy.

### Cationic halogen‐bond donors

As *Ω*_σ*_ performed surprisingly well even for quite challenging neutral test molecules, we further challenged its performance by employing cationic halogen‐bond donors as substrates. Although neutral polyfluorinated halogen‐bond donors predominate in the field of crystal engineering, cationic heteroarene‐based compounds like iodoimidazolium or iodotriazolium derivatives are frequently used in solution‐phase applications of halogen bonding. It should be stressed that these compounds feature very different σ‐hole depths and σ* orbital levels, and because the test set was entirely comprised of neutral halogen‐bond donors, a perfect correlation with the same linear combination of σ‐hole and σ* orbital would be somewhat surprising.

**Figure 23:**
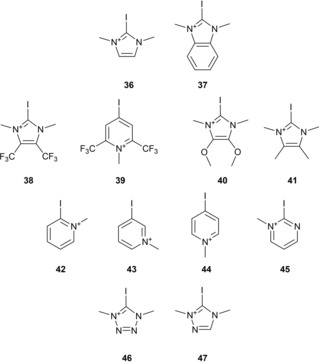
Set of cationic species (cat) **36**–**47**.

**Figure 24:**
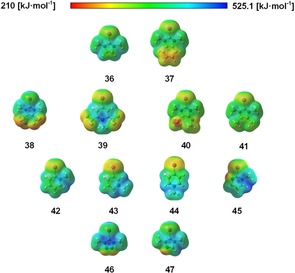
Electrostatic potentials for cationic compounds **36**–**47** computed at the 0.001 electron Bohr^−3^ isodensity surface, plotted in the potential range 210–525.1 kJ mol^−1^. Note that 210 kJ mol^−1^ is approximately the *v*_s,max_ value for iodocyanide (**18**), which is the most positive point in the test set and here the smallest positive value.

The set of cationic species (cat, Figure 23) includes four iodoimidazolium derivatives **36**, **38**, **40** and **41** with different substitution patterns, one benzimidazolium compound **37** and the triazolium **47** as well as tetrazolium derivative **46**. In addition, iodopyrimidinium **45** and four iodopyridiniums, **39** and **42**–**44**, were included. The latter feature *o*‐, *m*‐ and *p*‐iodination as well as a particularly electron‐deficient compound. The electrostatic potentials of all the compounds are shown in Figure 24, with the most positive point of iodocyanide (210 kJ mol^−1^) acting as reference.

**Figure 25:**
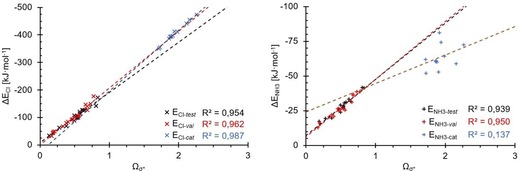
Plots of *Ω*_σ*_ versus the binding energies of the neutral halogen‐bond donors (red/black) and the cationic species (blue). Although all three datasets for chloride (left) show good correlations, this is not the case for ammonia (right); the data points for the cationic donors are scattered, with a low *R* ^2^ value.

The correlation between the binding energies of the chloride and ammonia complexes and the *Ω*_σ*MO_ parameter is shown for both the neutral molecules (test set (black) and validation set (red)) and the cationic species (blue) in Figure 25. For the ammonia data, it is immediately evident that there is no overlap of *Ω*_σ*_ or the binding energy values between the complexes involving neutral or charged halogen‐bond donors. Furthermore, and even more importantly, the data points for the cations are almost completely uncorrelated, featuring an *R* ^2^ value of only 0.14. On the other hand, and in complete contrast, the *R* ^2^ value for the chloride associations is slightly higher (0.99) than the ones of the neutral compounds, and furthermore, the regressions are almost collinear. Thus, overall, a re‐optimisation of the *a* and *b* coefficients for cationic halogen‐bond donors seemed necessary. Prior to this, we were also curious about the performance of *Ω*_σ*MO_ for non‐C−I‐based halogen‐bond donors.

**Figure 26:**
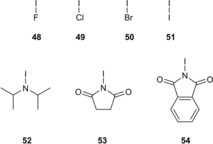
Four interhalogen compounds (**48**–**51**) and three N−I compounds (**52**–**54**) as model halogen‐bond donors.

**Figure 27:**
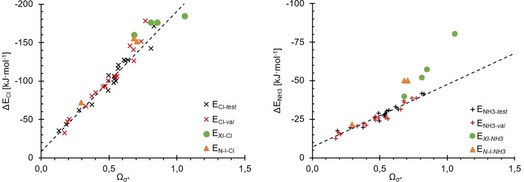
Addition of computed *Ω*_σ*_ for the interhalogens **48**–**51** (green dots) and organic N−I compounds **52**–**54** (orange triangles) to the data for the test and validation sets with *Ω*_σ*_ on the *x* axis and association energies for chloride and ammonia on the *y* axis.

Correspondingly, *Ω*_σ*_ values were computed for the four interhalogen compounds **48**–**51** and the three N−I compounds **52**–**54** (Figure 26). This comparatively small number of compounds should allow a first impression while still keeping the computational effort reasonable. For the chloride complexes, none of the seven compounds seem very far off the linear correlation, even though the interhalogen compounds clearly feature a different slope (green points, Figure 27, left).

The data points for the ammonia complexes also show different slopes for the N−I compounds (orange points, Figure 27, right) and particularly for the interhalogens (green points, Figure 27). Furthermore, both types of complexes show a quite substantial deviation from the linear regression line of the test and validation complexes.

So overall, neither the “inorganic” interhalogen compounds nor the N−I‐based halogen‐bond donors can be treated with the same linear combination parameter *Ω*_σ*_ as the previously mentioned neutral C−I‐based Lewis acids. Because this study was primarily intended for carbon‐based halogen‐bond donors, we will not investigate this aspect further.

**Figure 28:**
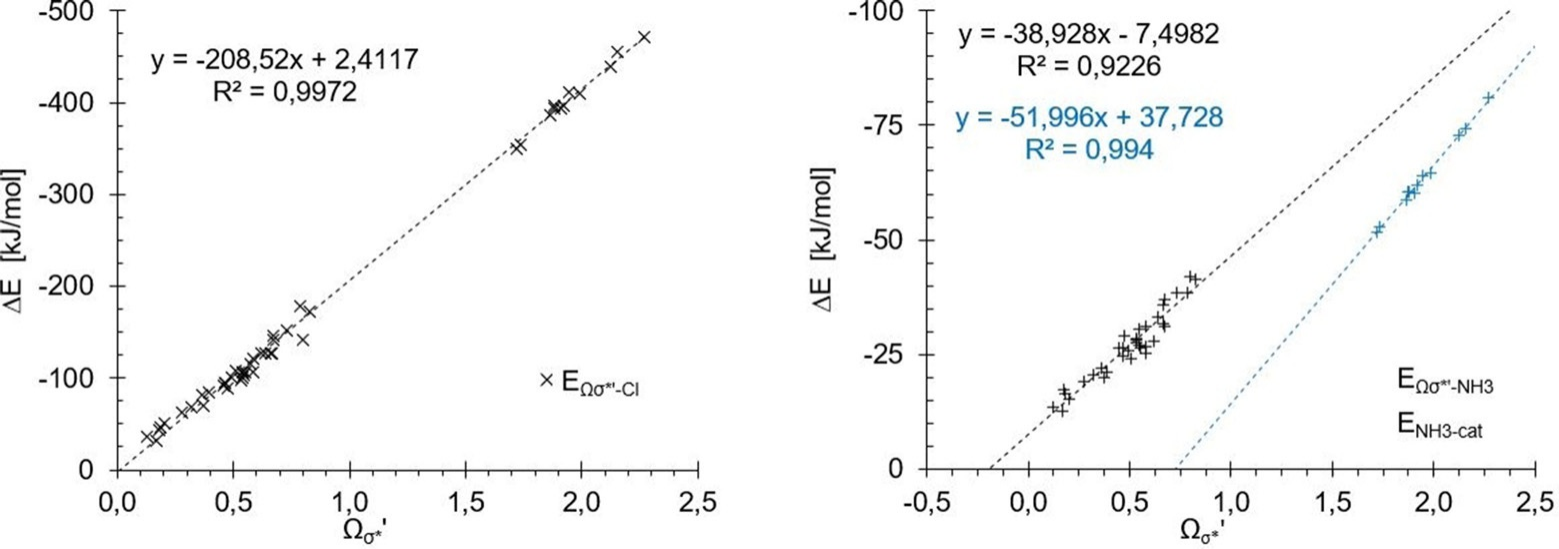
Results of the re‐optimisation with *Ω*_σ*_′ that includes the test, validation and cation sets. Chloride complexes (left) with one overall linear regression and ammonia complexes (right) with one regression for the test and validation sets (black) and one for the *cationic* set (blue).

In contrast, a re‐optimisation of *Ω*_σ*_ was attempted that would also consider the data points of the cationic (cat) set of halogen‐bond donors (in addition to the test and validation sets). Good results were obtained with the parameters *a*=3.39×10^−3^ and *b*=1.05×10^−3^ [see Eq. (1)] and they are represented graphically in Figure 28. This re‐optimised parameter will be denoted *Ω*_σ*_′ from here on.

As can be seen in the left‐hand graph of Figure 28, the combination of the binding data of all three donor sets results in an almost perfect linear regression (*R* ^2^=0.99) for the chloride complexes. Notably, the correlation of *Ω*_σ*_′ with the binding energies of the cationic halogen‐bond donors is much improved compared with before. The same is true for the ammonia complexes, and a linear regression over all data points results in a very good correlation (*R* ^2^=0.96). However, visual inspection of the graph (Figure 28, right) clearly indicates that there are actually two different trend lines, one for the test and validation sets (black) and one for the cationic compounds (blue). The correlation coefficient for the former is now only slightly worse than previously (*R* ^2^=0.92 for *Ω*_σ*_′ compared with *R* ^2^=0.93/0.94 for *Ω*_σ*_), whereas the correlation for the cationic compounds is almost perfect (*R* ^2^=0.99 for *Ω*_σ*_′ compared with *R* ^2^=0.14 for *Ω*_σ*_).

**Figure 29:**
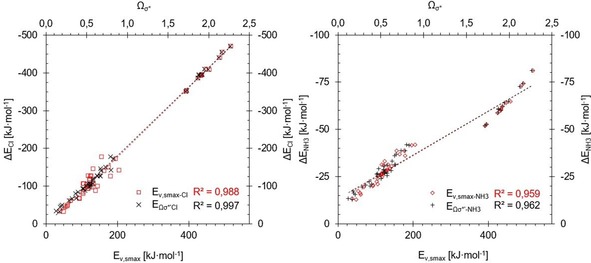
Overlay of the computed *v*_s,max_ energies (lower *x* axes, red) on the generated *Ω*_σ*_′ (upper *x* axes, black) values for all compounds **1**–**47** plotted against the calculated association energies with chloride (left) and ammonia (right). All *R* ^2^ values are above 0.95, but for subsets with smaller ranges of energies, for example, neutral XB donors with chloride, *Ω*_σ*_′ reproduces the energy trend far better than the σ‐hole alone (see below).

Finally, we compared our new parameter *Ω*_σ*_′ directly with *v*_s,max_ and plotted an overlay of the correlations of both to the binding energies (Figure 29). Correlation coefficients close to 1.0 were found for both parameters for the chloride complexes, and almost identical values of 0.96 for the ammonia complexes.^48^ Thus, at first glance, both parameters seem to perform almost identically. However, this may in part be due to the relatively large range of energies considered, because when trends within narrower energy ranges are investigated (e.g., neutral XB donors with *v*_s,max_ values in the range 100–150 kJ mol^−1^), trends are predicted much better by *Ω*_σ*_′ than by *v*_s,max_.

**Figure 30:**
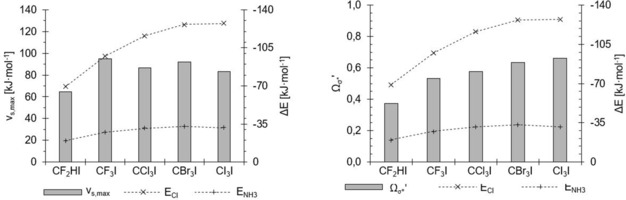
Comparison of *v*_s.,max_ (left) and *Ω*_σ*_′ (right) (*y* axes on left, grey bars) of the halomethanes **1**–**5** (*x* axes) with their association energies with chloride (×) and ammonia (+) (*y* axes on right), sorted according to increasing Cl^−^ association energy.

**Figure 31:**
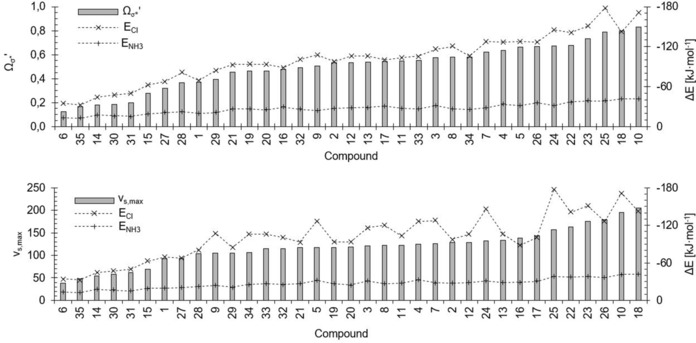
Comparison of *v*_s,max_ (bottom) and *Ω*_σ*_′ (top) (*y* axes on left, grey bars) of the test set compounds **1**–**18** (*x* axes) with their association energies with chloride (×) and ammonia (+) (*y* axes on right), sorted according to increasing *v*_s,max_ and *Ω*_σ*_′, respectively.

This is illustrated in different ways in Figures 30 and 31. In Figure 30, compounds are ordered according to increasing Lewis acidity from left to right (with chloride and ammonia binding energies shown as dashed lines) and the corresponding *Ω*_σ*_′ or *v*_s,max_ values represented as bars. Figure 30 focuses on the subset of halogen‐bond donors investigated previously,^45^ and the graph on the left (identical to Figure 3) demonstrates that the trend in Lewis acidity within this subset is not at all reproduced by *v*_s,max_ values. As the graph on the right clearly illustrates, however, the *Ω*_σ*_′ values echo the Lewis acidity very nicely.

In Figure 31, the neutral XB donors are ordered according to ascending *Ω*_σ*_′ (top) or *v*_s,max_ values (bottom). In the case of the former, the trend in binding energies, particularly for the chloride complexes, is mostly in line with a gradual increase from left to right, with very few outliers. In the case of the σ‐hole, the corresponding trend lines for the binding energies seem much more erratic.

## Conclusion and Outlook

The correlations between various potential parameters for halogen‐bond‐based Lewis acidity and the (computed) gas‐phase binding energies of these Lewis acids to chloride and ammonia (as typical Lewis bases) have been evaluated. The best single parameter for predicting halogen‐bonding strength is the σ‐hole depth (*v*_s,max_), which is especially useful in the description of weak (neutral) halogen‐bonded complexes, but shows some shortcomings in the prediction of adducts featuring an anionic Lewis base.

To obtain a better indicator for the prediction of Lewis acidity, linear combinations of individual parameters were then tested in a completely empirical approach. The linear combination of the σ‐hole depth with the energy of the σ* orbital, suitably optimised against a test set of halogen‐bond donors (**1**–**18**), was found to be superior in the prediction of halogen‐bonding strength, especially for the stronger complexes. This parameter *Ω*_σ*_ provided a strong correlation when validated against a second set of diverse neutral halogen‐bond donors (**19**–**35**). Cationic halogen‐based Lewis acids were described with a high accuracy for chloride association and a very low accuracy for ammonia by this parameter, but a re‐optimisation led to the derivative *Ω*_σ*_′, which once again provided a strong correlation with the gas‐phase binding energies.

The advantages of *Ω*_σ*_′ compared with single parameters like the σ‐hole depth is visualised by means of a colour code in Table 3. For each halogen‐bonded complex, the binding energy (*E*_Cl_ and *E*_NH3_) is given but is also depicted in a colour ranging from red (weak) to blue (strong). For comparison, the values of the σ‐hole depth (*v*_s,max_), σ* orbital energy and *Ω*_σ*_′ are presented on the same line for each complex, using a similar colour code.

**Table 3:** Association energies of compounds **1**–**18** (sorted according to increasing bond strength) with chloride (left) and ammonia (right) in direct comparison with the *v*_s,max,_, σ* orbital energies and our newly defined *Ω*_σ*_′. Higher values are highlighted in blue, medium in white and lower in red.

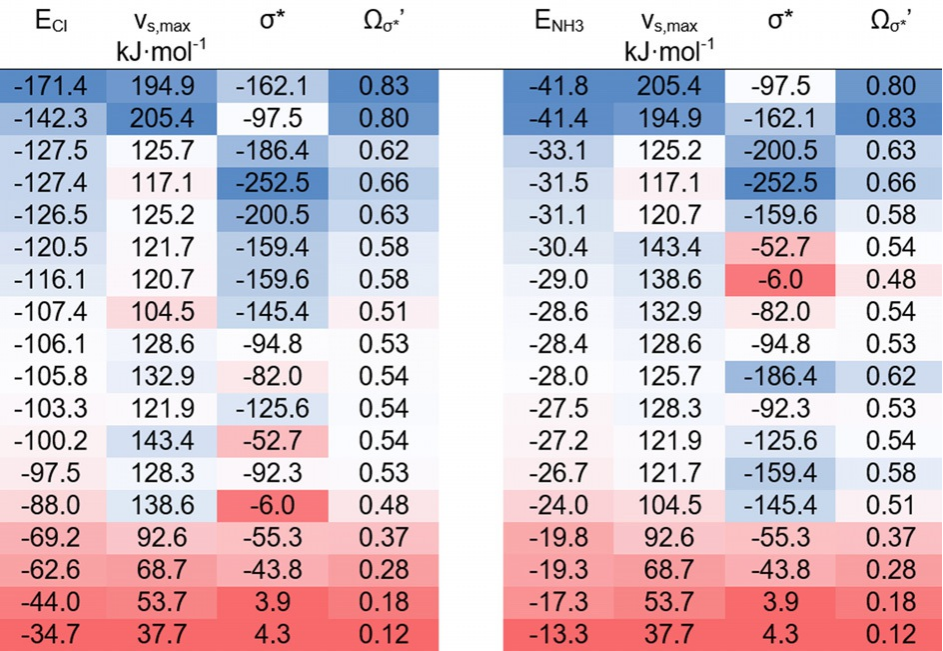

Although the σ*orbital energies are clearly not useful for predicting the relative order of halogen‐bonding strength (as is apparent by the many deviations in the colours of the entries), neither do the σ‐hole values reproduce the actual ranking in a clean fashion (see, for example, the light‐blue/white entries in the lower half of Table 3). The combined parameter, however, yields an almost exact ordering of the relative halogen‐bonding capabilities of the iodinated compounds.

With *Ω*_σ*_ as a novel tool in hand, we will now parametrise further classes of halogen‐bond donors. Ideally, this will lead, on the one hand, to a better a priori prediction of the suitability of specific halogen‐bond donors for various applications (including catalysis). On the other hand, it may also lead to a deeper understanding of the relative contributions of electrostatics and “charge transfer” (and other components) to the overall halogen‐bonding interaction energy. Thus, the fact that different “mixing ratios” are required for different types of halogen‐bond donors (e.g., C−I‐ vs. N−I‐based compounds) may provide a coarse diagnostic tool for the relative importance of pure electrostatics for their halogen‐bonding interactions.

## Experimental Section

All DFT calculations and MP2 optimisations (without counterpoise correction) were performed by using Gaussian 09.23b, 23c, 37b, 37c, 49 We employed the B3LYP, B97D3, M05‐2X, M06‐2X, mPW1PW91 and ωB97xD functionals with and without additional Grimme (D3) dispersion correction on the def2‐SVP(D), def2‐TZVP, def2‐TZVP(D) and def2‐TZVPP basis sets.^26^, 27a, 28, 29, 30, 32, 33 Symmetry was applied whenever possible and only turned off when persistent imaginary frequencies could not be otherwise overcome, which was mostly the case for complexes with ammonia. Gaussian output files were analysed with GoodVibes,^50^ molecular orbitals and electrostatic potentials were plotted by using GaussView.^51^ Harmonic vibrational frequency scaling factors were taken from the work of Truhlar^52^ and Martin^53^ and their co‐workers, or calculated according to the latter.^54^ Frequencies below 100 cm^−1^ were treated according to the approaches of Truhlar and co‐workers^55^ and Grimme.^56^ Correlation factors (*Ω*) were calculated by using the Excel solver tool. Coupled cluster (CCSD(T)) energies with the def2‐TZVPPD and def2‐QZVPPD basis sets were calculated by using Turbomole^57^ and those with Dunning basis sets by using Molpro.^58^ Complete basis set limits were obtained by the methods of Feller extrapolating over three cardinal numbers (2–4) when Dunning basis sets were employed.^38^ For the cases of the Karlsruhe basis sets, the contributions of higher‐order correlation energies were determined by extrapolating the MP2 energies and correlating them with the CCSD(T)/def2TZVPPD energies.39b, 59

## Conflict of interest

The authors declare no conflict of interest.

## Supporting information

As a service to our authors and readers, this journal provides supporting information supplied by the authors. Such materials are peer reviewed and may be re‐organized for online delivery, but are not copy‐edited or typeset. Technical support issues arising from supporting information (other than missing files) should be addressed to the authors.

Supplementary

Supplementary
