# Supplementary material for: Is There a Single Ideal Parameter for Halogen‐Bonding‐Based Lewis Acidity?
Source: Chemistry. 2020 Mar 9;26(17):3843–61. doi: 10.1002/chem.201905273 (PMC7154672; doi:10.1002/chem.201905273)
Supplement: Supplementary file 1 — Supplementary [file CHEM-26-3843-s001.pdf]

CHEMISTRY
A European Journal

Supporting Information

Is There a Single Ideal Parameter for Halogen-Bonding-Based Lewis Acidity?

Elric Engelage, Dominik Reinhard, and Stefan M. Huber*[a]

chem_201905273_sm_XBcorrelationb.xlsx
